# Oral manifestations of HIV infection in children and adults receiving highly active anti-retroviral therapy [HAART] in Dar es Salaam, Tanzania

**DOI:** 10.1186/1472-6831-6-12

**Published:** 2006-08-18

**Authors:** Omar JM Hamza, Mecky IN Matee, Elison NM Simon, Emil Kikwilu, Mainen J Moshi, Ferdinand Mugusi, Frans HM Mikx, Paul E Verweij, André JAM van der Ven

**Affiliations:** 1 Department of Oral Surgery and Oral Pathology, Muhimbili University College of Health Sciences, Dar es Salaam, Tanzania; 2 Department of Microbiology and Immunology, Muhimbili University College of Health Sciences, Dar es Salaam, Tanzania; 3 Department of Preventive and Community Dentistry, Muhimbili University College of Health Sciences, Dar es Salaam, Tanzania; 4 Institute of Traditional Medicine, Muhimbili University College of Health Sciences, Dar es Salaam, Tanzania; 5 Department of Internal Medicine, Muhimbili University College of Health Sciences, Dar es Salaam, Tanzania; 6 WHO Collaborating Center, Dentistry, Radboud University Nijmegen Medical Center, Nijmegen, The Netherlands; 7 Department of Medical Microbiology, Radboud University Nijmegen Medical Center, Nijmegen, The Netherlands; 8 Department of General Internal Medicine, Radboud University Nijmegen Medical Center, Nijmegen, The Netherlands

## Abstract

**Background:**

The aim of the study was to compare the prevalence and types of HIV-related oral lesions between children and adult Tanzanian patients on HAART with those not on HAART and to relate the occurrence of the lesions with anti-HIV drug regimen, clinical stage of HIV disease and CD4+ cell count.

**Methods:**

Participants were 532 HIV infected patients, 51 children and 481 adults, 165 males and 367 females. Children were aged 2–17 years and adults 18 and 67 years. Participants were recruited consecutively at the Muhimbili National Hospital (MNH) HIV clinic from October 2004 to September 2005. Investigations included; interviews, physical examinations, HIV testing and enumeration of CD4+ T cells.

**Results:**

A total of 237 HIV-associated oral lesions were observed in 210 (39.5%) patients. Oral candidiasis was the commonest (23.5%), followed by mucosal hyperpigmentation (4.7%). There was a significant difference in the occurrence of oral candidiasis (χ^2 ^= 4.31; df = 1; p = 0.03) and parotid enlargement (χ^2 ^= 36.5; df = 1; p = 0.04) between children and adults. Adult patients who were on HAART had a significantly lower risk of; oral lesions (OR = 0.32; 95% CI = 0.22 – 0.47; p = 0.005), oral candidiasis (OR = 0.28; 95% CI = 0.18 – 0.44; p = 0.003) and oral hairy leukoplakia (OR = 0.18; 95% CI = 0.04 – 0.85; p = 0.03). There was no significant reduction in occurrence of oral lesions in children on HAART (OR = 0.35; 95% CI = 0.11–1.14; p = 0.15). There was also a significant association between the presence of oral lesions and CD4+ cell count < 200 cell/mm^3 ^(χ^2 ^= 52.4; df = 2; p = 0.006) and with WHO clinical stage (χ^2 ^= 121; df = 3; p = 0.008). Oral lesions were also associated with tobacco smoking (χ^2 ^= 8.17; df = 2; p = 0.04).

**Conclusion:**

Adult patients receiving HAART had a significantly lower prevalence of oral lesions, particularly oral candidiasis and oral hairy leukoplakia. There was no significant change in occurrence of oral lesions in children receiving HAART. The occurrence of oral lesions, in both HAART and non-HAART patients, correlated with WHO clinical staging and CD4+ less than 200 cells/mm^3^.

## Background

Most African studies on HIV associated oral lesions have been done during pre-HAART era [1-11]. The relatively few studies of oral lesions in patients on HAART have been conducted elsewhere and do indicate significant differences in the influence of HAART on types of oral lesions [12-14]. For example, oral candidiasis, oral hairy leukoplakia, Kaposi's sarcoma and HIV-associated periodontal diseases have been reported to decrease [12,13,15-22]. On the other hand, HIV-salivary gland disease, human papilloma virus (HPV)-associated oral lesions including papilloma, condylomas and focal epithelial hyperplasia [oral warts], xerostomia and recurrent oral ulceration appear to have increased [13-15,18,23-25]. There are also reports indicating no change in the occurrence of HIV associated oral lesions in children receiving HAART [26,27]. The reasons for these differences are not entirely clear. Some authors have associated these variations with differences in access to oral health care, demographic and social factors, mode of HIV transmission, types of co-infections, disease stage and immune reconstitution [18,28,29]. Nonetheless, the presence of such significant differences underlines the need for meticulous monitoring of prevalence and types of HIV associated oral lesions in every clinical setting that provide HAART [13].

As indicated earlier, most of the above-mentioned studies have been conducted in developed countries [30,31]. There is relatively little information emanating from developing countries, where particular efforts are being made to scale up provision of HAART to eligible patients [32].

The government of Tanzania, with assistance from international donor agencies, started providing HAART to HIV patients free of charge in July 2004 [33,34]. However, there is no information regarding human immunodeficiency virus (HIV)-related oral lesions of patients on antiretroviral therapy. This study aimed to determine the prevalence and types of oral manifestations of HIV/AIDS between children and adult patients on HAART and those not on HAART, and correlate clinical oral lesions with clinical stage of HIV disease, anti-HIV drug regimen and CD4+ cell count.

## Methods

### Participants and setting

A total of 532 HIV-infected patients, 367 (69%) females and 165 (31%) males participated in the study. There were 51 children aged between 2 and 17 years, with mean age of 7.6 (SD ± 4.3) years and 481 adults aged 18–67 years, with a mean age of 38.2 (SD ± 8.9) years. Participants were recruited consecutively at the Muhimbili National Hospital (MNH) HIV clinic in Dar es Salaam, from October 2004 to September 2005. The MNH is the largest referral hospital in Tanzania and serves as a teaching facility for the Muhimbili University College of Health Sciences (MUCHS), the largest medical school in the country.

### Study design and sample size calculations

This was a cross sectional study. The sample size was estimated at 336, by assuming the prevalence of oral lesions in HIV infected individuals in Tanzania to be around 30% [4] and by setting type I error at 5% and type II error at 20%. We consecutively enrolled 532 patients. None of the patients declined participation in this study, bringing the participation rate to 100%.

### Investigations

Patients were interviewed using a standard structured questionnaire to obtain information regarding social and demographic details, past medical history, family history and history of previous medication. Previous episodes of oral candidiasis, other medical conditions, use of traditional medicine, anti-tuberculosis drugs, antifungal agents, and use of antiretroviral and current treatments were all recorded. Current and previous episodes of opportunistic systemic diseases related to HIV were categorized and recorded as follows: tuberculosis, pneumonia, herpes zoster infection and cryptococcal meningitis.

### General examination

Clinical examination of all study patients was done by an independent physician, who categorized them in accordance with the World Health Organization (WHO) clinical staging criteria [35].

### Oral examination

An oral examination was carried out by a qualified dental surgeon without knowing the HIV clinical stage and CD4+ cell count level of the patient or whether the patient was on HAART or not. A standard oral examination method recommended by WHO [36] was used to examine: (a) the extra-oral, head and neck areas; and (b), peri-oral and intra-oral soft tissues using a criteria described by Greenspan et al. [37]. Examination was conducted while the patient was seated on a chair under artificial light. The extra-oral and peri-oral tissues were examined first, followed by the intra-oral tissues, for changes in size, colour and shape of anatomical areas as well as for clinical signs and lesions. The oral lesions associated with HIV infection were diagnosed based on their clinical presentation and where multiple sites were involved, all sites were documented.

### HIV serology and determination of lymphocytes subsets

Blood was collected in EDTA tubes from the patients. HIV serology was determined by Vironostika HIV Uni-Form II Ag/Ab (BioMerieux, Boxtel, The Netherlands) and reactive samples were retested by Vironostika HIV Uni-Form II Plus O (BioMerieux, Boxtel, The Netherlands). Samples reactive on both tests were considered to be positive for IgG anti HIV antibodies. Enumeration of CD4+ and CD8+ T cells was done using FACS count machine after staining patients' blood with monoclonal antibodies [38].

### HAART regimens

In Tanzanian setting, a triple therapy consisting of 2 nucleoside reverse transcriptase inhibitors (NRTI) + 1 non-nucleoside reverse transcriptase inhibitors (NNRTI) or 2 NRTI + 1 Protease inhibitor (PI) is recommended. The first line includes four different combinations of drugs: stavudine + lamivudine + nevirapine; stavudine + lamivudine + effavirenz; zidovudine + lamivudine + nevirapine and zidovudine + lamivudine + effavirenz. The second line regimen includes the following drug combination: Abacavir + kaletra (lopinavir/ritonavir) + didanosine and abacavir + saquinavir/ritonavir + didanosine [34].

### Ethical issues

The study protocol was approved by the ethics committees of the Muhimbili University College of Health Sciences and Muhimbili National Hospital, Dar es salaam, Tanzania. Informed verbal consent was sought from participants and from parents/or guardians in case of children below eighteen years. The following information was given to ensure that patients and parents/guardians have the information needed to make an informed choice: a complete description of the aims of the study, potential benefits and risks, blood collection procedures and assurance of confidentiality of any information given as well as test results. Study personnel provided any other requested additional information. All patients seen in this study received appropriate care and treatment according to national guidelines on care and treatment of HIV infected individuals. Patients who were found to have any oral lesions were referred to the Department of Oral Surgery and Oral Pathology of the Muhimbili National Hospital where appropriate management and follow-up were given. All patients' information and test results were confidentially kept.

### Statistical analysis

Data were coded, entered, cleaned, validated and analyzed using the SPSS version 12.0 [39]. Patients were categorized into children (<18 years) and adults (≥18 years) and into those on HAART and those not receiving HAART. Comparison of proportions was performed using the Pearson chi-squared test and in situation where 20% or more of the cells had expected count less than 5, Fisher's exact test was used. Bivariate analysis was done using Spearman's rank correlations. A P-value of < 0.05 was considered significant. The following were determined: degree of associations between occurrence and types of oral lesions and use of anti-retrovirals, gender, smoking habits, alcohol consumption, coexistence with opportunistic systemic diseases, clinical stage of HIV disease and degree of immunosuppression. Multiple logistic regression analysis was performed to assess the association between presence of oral lesion associated with HIV and use of antiretrovirals after adjusting for CD4+ cell count.

## Results

A total of 532 HIV-infected patients, 367 (69%) females and 165 (31%) males participated in the study (Table 1). There were 51 children aged between 2 and 17 years, with mean age of 7.6 (SD ± 4.3) years and 481 adults aged 18–67 years, with a mean age of 38.2 (SD ± 8.9) years. Overall, median CD4+ cell count was 151 cells/mm^3^, with values ranging from 1 to 2007 cell/mm^3^. The median CD4+ cell count for children was 501 cells/mm^3 ^and 134 cells/mm^3 ^for adults. Majority 373 (70.1%) of children and adult patients were in the WHO clinical stages II to III. Among the participants, 298 (56%) patients were on HAART, consisting of nucleoside reverse transcriptase inhibitors (NRTIs), non-nucleoside reverse transcriptase inhibitors (NNRTIs) and protease inhibitors (PIs). Majority 531 (99.7%) were on the first line HAART combination, of whom 229 (76.8%) were on a combination of Stavudine/Lamivudine/Nevirapine, 32 (10.7%) on a combination of Zidovudine/Lamivudine/Effavirenz, 26 (8.7%) on Stavudine/Lamivudine/Effavirenz and 10 (3.4%) were on Zidovudine/Lamivudine/Nevirapine. One patient (0.3%) was on second line combination that included protease inhibitors (Abacavir/Lopinavir/Didanosine). Two hundred and thirty four (44%) patients were not receiving HAART. Sixty-two patients (11.7%) reported use of traditional medicines, of whom 36 (58%) were concomitantly using traditional medicines with HAART.

**Table 1:** Socio-demographic, treatment, WHO clinical stage and CD4 cell counts of study participants

**Characteristics**	**All patients n (%)**	Patients with oral lesions n (%)*	**χ^2^-value**	p-value**
**Gender**				
Male	165 (31.0)	63 (38.2)	0.17	0.68
Female	367 (69.0)	147 (40.1)		
**Age (Years)**				
Children 2–17	51 (9.6)	21 (41.2)	0.68	0.88
Adults 18 – 67	481 (90.4)	189 (39.3)		
**Smoking habit**				
Current	20 (3.8)	14 (70)	8.17	0.02
Stopped	17 (3.2)	7 (41.2)		
Never	495 (93.0)	189 (38.2)		
**Alcohol consumption**				
Current	31 (5.8)	10 (32.3)		
Stopped	37 (7.0)	16 (43.2)	0.90	0.64
Never	464 (87.2)	184 (39.6)		
**Traditional medicine**				
Yes	62 (11.7)	25 (40.3)	0.17	0.68
No	470 (88.3)	183 (38.9)		
**Antiretroviral therapy**				
Without therapy	234 (44.0)	127 (54.3)	38.30	0.00
HAART therapy	298 (56.0)	83 (27.9)		
**Antiretroviral types**				
Stav/Lamiv/Nev	229 (76.8)	9 (12.9)	2.96	0.57
Stav/Lamiv/Eff	26 (8.7)	10 (38.5)		
Zido/Lamiv/Eff	32 (10.7)	11 (34.4)		
Zido/Lamiv/Nev	10 (3.4)	2 (20.0)		
Abac/Lop/Didan	1 (0.3)	0 (0)		
**WHO HIV clinical stage**				
Stage I	70 (13.2)	9 (12.9)		
Stage II	204 (38.3)	38 (18.6)	120.93	0.00
Stage III	169 (31.8)	101 (59.8)		
Stage IV	89 (16.7)	62 (69.7)		
**CD4^+ ^cell count (cells/mm^3^)**				
> 500	46 (8.6)	10 (21.7)		
200–500	164 (30.8)	33 (20.5)	52.45	0.00
< 200	322 (60.5)	167 (31.9)		
**Total number of patients**	**532 (100.)**	**210 (39.5)**		

* Row percentages; ** p-value less than 0.05 was considered significant; χ^2 ^Chi square test

A total of 237 HIV-associated oral lesions were observed in 210 (39.5%) patients. Overall, oral candidiasis was the commonest oral lesion seen in 125 (23.5%) patients followed by mucosal hyper pigmentation 25 (4.7%), parotid gland enlargement 21 (3.9%) and oral Kaposi's sarcoma 17 (3.2%) (Table 2). In children, parotid gland enlargement was the commonest oral lesion (19.6%), followed by oral candidiasis (11.8%), oral Kaposi's sarcoma (3.9%), oral hairy leukoplakia (3.9%), herpes simplex lesions (2.2%) and oral warts was the least (2.0%). In adults, oral candidiasis was the commonest (24.7%), followed by mucosal hyperpigmentation (5.2%), herpes zoster face and odontogenic abscess was the least (0.4% each). There was statistically significant difference in the occurrence of oral candidiasis (X^2 ^= 4.31; df = 1; p < 0.05) and parotid enlargement (X^2 ^= 36.5; df = 1; p < 0.05) between children and adults. Pseudomembranous candidiasis was the most predominant type of oral candidiasis 66.4% (83/125) followed by a combination of pseudomembranous and erythematous 12% (15/125), erythematous candidiasis 9.6% (12/125), angular cheilitis 3.2% (4/125), combination of angular cheilitis with erythematous and hyperplastic variant were the least (1.6% each). Among 125 patients diagnosed with oral candidiasis 42 (33.6%) patients had previous history of oral candidiasis, 37 (29.6%) patients presented with esophageal symptoms such as dysphagia, odynophagia and chest pain at the time of examination.

**Table 2:** Occurrence of HIV-associated oral lesions among children and adults

**Oral lesion**	**Children (2–17 yrs) n (%)**	**Adults (18–67 yrs) n (%)**	**Total (n = 532) n (%)**
**Oral candidiasis**	6 (11.8)	119 (24.7)	125 (23.5)
**Hyperpigmentation**	0 (0)	25 (5.2)	25 (4.7)
**Enlarged Parotid gland**	10 (19.6)	11 (2.3)	21 (3.9)
**Oral Kaposi's sarcoma**	2 (3.9)	15 (3.1)	17 (3.2)
**Necrotizing Ulcerative gingivitis**	0 (0)	13 (2.7)	13 (2.4)
**Oral hairy leukoplakia**	2 (3.9)	10 (0.6)	12 (2.3)
***Herpes simplex *lesions**	7 (2.2)	2 (2.1)	9 (1.7)
**Recurrent ulcers**	0 (0)	4 (0.8)	4 (0.8)
**Oral warts**	1 (2.0)	3 (0.6)	4 (0.8)
**Bell's palsy**	0 (0)	3 (0.6)	3 (0.6)
***Herpes zoster *face**	0 (0)	2 (0.4)	2 (0.4)
**Odontogenic abscess**	0 (0)	2 (0.4)	2 (0.4)
**All oral lesions**	**21 (41.2)**	**189 (39.3)**	**210 (39.5)**

Table 3 shows no significant difference in prevalence of oral lesions among children on HAART and those not on HAART (OR = 0.35; 95% CI = 0.11 – 1.15; p > 0.05). Table 4 shows that adult patients who were on HAART had a significantly lower risk of oral lesions (OR = 0.32; 95% CI = 0.22–0.47; p < 0.01), oral candidiasis (OR = 0.28; 95% CI = 0.18 – 0.44; p < 0.01) and oral hairy leukoplakia (OR = 0.18; 95% CI = 0.04 – 0.85; p < 0.03). There was also lower prevalence of necrotizing ulcerative gingivitis, herpes simplex lesions, recurrent ulcers, Bell's palsy and herpes zoster of face in adult patients who were on HAART, but the difference was not statistically significant (p > 0.05). After controlling for CD4+ cell count, adult on HAART had a significantly lower risk of oral Kaposi's sarcoma compared with those not on HAART (adjusted OR = 0.29; 95% CI = 0.10–0.89; p < 0.03). The odds for oral warts and mucosal hyper pigmentation were non-significant higher in adult who were on HAART, being (OR = 1.49; 95% CI = 0.13 – 16.5; p > 0.05) and (OR = 1.62; 95% CI = 0.68 – 3.82; p > 0.05), respectively.

**Table 3:** Comparison of oral lesions among children on HAART (n = 22) and those not on HAART (n = 29)

**Type of oral lesions**	**No. of oral lesions n (%)**	**Unadjusted OR (95% CI)**	**Adjusted OR (95% CI)**	**p-value**
**Oral candidiasis**				
On HAART	0 (0.0)	0.0 (0.0–9.20)	0.0 (0.0–1.80)	0.80
Not on HAART	6 (20.7)			
**Enlarged Parotid gland**				
On HAART	4 (18.2)	0.8 (0.2–3.48)	0.9 (0.22–3.93)	0.82
Not on HAART	6 (20.7)			
**Oral Kaposi's sarcoma**				
On HAART	1 (4.5)	1.3 (0.08–22.6)	0.9 (0.05–17.4)	0.84
Not on HAART	1 (3.4)			
**Oral hairy leukoplakia**				
On HAART	0 (0.0)	0.0 (0.0–2.61)	0.0 (0.0–2.11)	0.88
Not on HAART	10 (6.9)			
**Herpes simplex lesions**				
On HAART	0 (0.0)	0.0 (0.0–1.52)	0.0 (0.0–7.30)	0.92
Not on HAART	1 (3.4)			
**Oral warts**				
On HAART	1 (4.5)	-	-	0.91
Not on HAART	0 (0.0)			

**Table 4:** Comparison of oral lesions among adults on HAART (n = 276) and those not on HAART (n = 205)

**Type of oral lesions**	**No. of oral lesions n (%)**	**Unadjusted OR (95% CI)**	**Adjusted OR (95% CI)**	**p-value**
**Oral candidiasis**				
On HAART	41 (14.9)	0.28 (0.18–	0.17 (0.11–0.28)	0.00
Not on HAART	78 (38.0)	0.44)		
**Hyper pigmentation**				
On HAART	17 (6.2)	1.62 (0.68–	1.60 (0.67–3.81)	0.27
Not on HAART	8 (3.9)	3.82)		
**Enlarged Parotid gland**				
On HAART	6 (2.2)	0.89 (0.27–	0.92 (0.27–3.10)	0.85
Not on HAART	5 (4.9)	2.95)		
**Oral Kaposi's sarcoma**				
On HAART	5 (1.8)	0.36 (0.12–	0.29 (0.10–0.89)	0.06
Not on HAART	10 (4.9)	1.06)		*(0.03)
**Necrotizing Ulcerative gingivitis**				
On HAART	5 (1.7)	0.45 (0.15–1.41)	0.37 (0.12–1.15)	0.17
Not on HAART	8 (3.4)			
**Oral hairy leukoplakia**				
On HAART	2 (0.7)	0.18 (0.04–	0.15 (0.03–0.70)	0.03
Not on HAART	8 (3.9)	0.85)		
**Herpes simplex lesions**				
On HAART	3 (1.1)	0.44 (0.10–	0.39 (0.09–1.69)	0.26
Not on HAART	5 (2.4)	1.86)		
**Recurrent ulcers**				
On HAART	1 (0.4)	0.24 (0.02–	0.22 (0.02–2.15)	0.22
Not on HAART	3 (1.54)	2.37)		
**Oral warts**				
On HAART	2 (0.7)	1.49 (0.13–	1.44 (0.13–16.4)	0.75
Not on HAART	1 (0.5)	16.5)		
**Bell's palsy**				
On HAART	0 (0.0)	-	-	0.84
Not on HAART	3 (1.5)			
**Herpes zoster face**				
On HAART	0 (0.0)	-	-	0.85
Not on HAART	2 (0.9)			
**Odontogenic abscess**				
On HAART	1 (0.4)	0.74 (0.04–11.9)	0.78 (0.05–13.0)	0.83
Not on HAART	1 (0.5)			

* p-value obtained after adjusting for CD4+ cell counts.

The duration of use of HAART ranged from 1 to 14 months, with majority (71.1%) having been on treatment for one to six months. Patients on HAART for the duration of more than six months had significantly lower prevalence of oral lesions (OR = 0.53; 95% CI 0.29–0.97; p < 0.05) and specifically oral candidiasis (OR = 0.53; 95% CI 0.29–0.97; p = 0.04).

The mean CD4+ profiles of patients on HAART and those not on HAART are shown in Table 5. Overall, the majority (67.8%) of patients who were on HAART had CD4^+ ^cell count < 200 cells/mm^3^. Only a small minority (32.2%) of the patients on HAART were found to have CD4^+ ^counts > 200 cells/mm^3^. Among those who were not on HAART 51.3% had CD4^+ ^cell count < 200 cells/mm^3 ^while the rest in the group had CD4^+ ^cell count > 200 cells/mm^3^. However, the majority (70.3%) of adult patients on HAART had CD4^+ ^cell count < 200 cells/mm^3 ^while only minority (36.4%) of the children on HAART had CD4^+ ^cell count < 200 cells/mm^3^. The mean difference of CD4+ cell counts between the adult and children on HAART and those not on HAART was statistically significant (t = 3.94; 95% CI = 42.3 – 126.3; p < 0.01). In both HAART and non-HAART receiving children and adult patients oral lesions occurred more significantly among those with CD4+ cell count less than 200 cell/mm^3 ^(χ^2 ^= 52.4; df = 2; p < 0.01). There was a strong association between the WHO clinical stage of HIV disease with the presence of oral lesions (χ^2 ^= 121; df = 3; p < 0.01), particularly oral candidiasis (χ^2 ^= 112; df = 3; p < 0.01), oral Kaposi's sarcoma (χ^2 ^= 121; df = 3; p < 0.01), and oral hairy leukoplakia (χ^2 ^= 10; df = 3; p < 0.05).

**Table 5:** CD4+ cell counts of Patients on HAART and those not on HAART

	**Patients on HAART (n = 298)**			**Patients without HAART (n = 234)**		
	**n (%)**	**Mean**	**SD**	**n (%)**	**Mean**	**SD**
**CD4+ cell count (cells/mm^3^)**						
**<200**	202(67.8)	84.0	62.1	120 (51.3)	65.2	57.0
**200–500**	80(26.8)	296.0	75.0	84 (35.9)	314.4	90.2
**>500**	16(5.4)	714.1	207.1	30 (12.8)	879.9	415.2
**Total**	**298**	**174.8**	**177.5**	**234 259.1**	**310.0**	**470**

Student's t-test for comparing means; (t = 3.94; 95% CI = 42.3 – 126.3; p = 0.00).

Eighty-four (15.8%) patients had current systemic diseases at the time of examination. Sixty-two (11.7%) patients had pulmonary tuberculosis (TB) and were on anti-tuberculosis treatment. Other systemic opportunistic diseases encountered at the time of examination were; pneumonia 14 (2.6%) patients and herpes zoster 8 (1.5%) patients. One hundred and thirty eight (25.9%) reported history of herpes zoster infection, 84 patients (15.8%) pneumonia while cryptococcal meningitis was reported by only 10 patients (1.9%). There was a significant association between occurrence of pulmonary TB with oral lesions (χ^2 ^= 6.9; df = 1; p < 0.01) and oral candidiasis (χ^2 ^= 11.0; df = 1; p < 0.01) but not other systemic diseases (P > 0.05). Oral lesions were not associated with any of the investigated socio-demographic features except for tobacco smoking (χ^2 ^= 8.17; df = 2; p < 0.01).

## Discussion

In the present study, the prevalence of HIV-associated oral lesions in adult patients receiving HAART was significantly reduced. The reduction was mainly attributed to the reduction of oral candidiasis and oral hairy leukoplakia among adult patients. This finding is consistent with observations in other studies that have shown significant reduction in these oral lesions associated with HAART usage, linked to the improved immune status of patients [12,13,15-22]. Notably, patients on HAART for the duration of more than six months had significantly lower prevalence of oral lesions (OR = 0.53; 95% CI 0.29–0.97; p < 0.05) and specifically oral candidiasis (OR = 0.53; 95% CI 0.29–0.97; p = 0.04). There was, however, no significant association between the type of HAART regimen and presence of oral lesions, most probably due to the fact that most of our patients (76.8%) were on one type of HAART consisting of stavudine, lamivudine and nevirapine, and that only one patient had a HAART combination containing PI. We found no significant difference in prevalence of oral lesions between children on HAART and those not on HAART (Table 3), similar to other studies [26,27]. The occurrence of HIV-associated oral in children is an issue that requires further investigation as the results of different studies have produced conflicting results. In one study there was no direct relationship between prevalence of oral lesions, severe immunodepression, and/or viral load > 100000 copies in children who were perinataly infected with HIV [40].

There were no significant difference in prevalence of oral lesions between adults (39.3%) and children (41.2%), (p > 0.05). However, a significantly higher prevalence of enlargement of parotid glands in children and oral candidiasis in adults was observed, which is in keeping with the findings of Greenspan et al. 1992 [37].

Mucosal hyperpigmentation, which was the second most prevalent oral lesions after oral candidiasis with a prevalence of 4.7%, occurred at higher, but non-significant magnitude, among adult patients who were on HAART. The reported prevalence of mucosal hyperpigmentation compares with a prevalence of 6% among HIV/AIDS patients in Kenya [9]. The higher prevalence of mucosal hyperpigmentation in patients on HAART has been linked with increased melanin production in the epithelium associated with increased release of á-melanocyte-stimulating hormone (á – MSH) [41] as a result of systemic ketoconazole and zidovudine therapy [42,43]. In the present study 20% (5/25) of patients with mucosal hyperpigmentation were on HAART combination containing zidovudine.

Likewise, there was insignificant increase in oral papillomata [oral warts] in patients on HAART as compared to the non-HAART group. The increase in oral warts among patients on HAART has been associated with immune reconstitution [18,23-25]. The lack of significant association between oral wart and HAART usage observed in the present study could be due to the short period of the administration of HAART as majority of patients were on HAART for one to six months.

There were no differences in the prevalence of other oral lesions including enlarged parotid glands, necrotizing ulcerative gingivitis, recurrent ulcers, Bell's palsy and herpes zoster of the face between HAART and non-HAART groups (p > 0.05). This could be explained by the fact that most of these lesions are classified as being less commonly associated with HIV [44] and therefore, their prevalence even in the non-HAART receiving group was small.

The use of traditional medicine among our patients was high (11.7%), which is approximately one in eight patients, an observation that is in line with that reported earlier in Tanzania [45]. Although no significant association was found between occurrences of oral lesions and use of traditional medicine (Table 1) there is still a need for further studies since many people with HIV/AIDS use traditional medicine in addition to HAART. It is crucial to learn more about the nature and composition of these plant medicines and their potential interactions with drugs that are used for management of HIV/AIDS and its associated opportunistic diseases.

In this study, 15.8% of patients diagnosed to have systemic diseases such as tuberculosis, pneumonia and herpes zoster at the time of examination. However, the only significant association found was between pulmonary TB and oral lesions, specifically oral candidiasis (p <= 0.01) (Table 6). This association possibly reflects immune deterioration in patients with oral candidiasis and TB. This finding, which is in keeping with that of Nittayananta et al. 2002 [46] who found significant association between the occurrence of TB and the presence of oral candidiasis (OR 2.8; 95% CI 1.6–4.8; *P *< 0.01), and positive predictive values of any oral lesions and oral candidiasis in predicting TB of 87% (95% CI 73.0–94.6) and 67% (95% CI 51.9–80.0), respectively seem to imply that oral candidiasis might be used as an additional clinical marker for TB together with clinical presentation of weight loss with night sweats and chest symptoms. Majority (25.9%) reported history of herpes zoster infection, 84 patients (15.8%) pneumonia while cryptococcal meningitis was reported by only 10 patients (1.9%). However, there was no statistically significant difference between prevalence of oral lesions and history of opportunistic systemic diseases (P > 0.05). The limitation on this part of the study was dependency on patients to recall previous opportunistic disease.

**Table 6:** Association in the occurrence of oral lesions and opportunistic systemic diseases among HIV-infected patients expressed as Odds ratios (OR) with 95% Confidence intervals

**Systemic disease**	**n (%)**	**OR (95% CI)**	**χ^2^-value (p-value)**
**Tuberculosis**	**62 (11.7)**		
All oral lesions	34 (54.8)	2.02 (1.19–3.46)	6.9 (0.008)
Oral candidiasis	25 (40.3)	2.50 (1.44–4.34)	11.0 (0.001)
**Pneumonia**	**14 (2.6)**		
All oral lesions	4 (28.6)	0.61 (0.19–1.96)	0.71 (0.40)
Oral candidiasis	2 (14.3)	0.54 (0.12–2.42)	0.68 (0.41)
**Herpes zoster**	**8 (1.5)**		
All oral lesions	2 (25.0)	0.51 (1.01–2.53)	0.71 (0.32)
Oral candidiasis	2 (25.0)	1.08 (0.22–5.45	0.01 (0.60)

Lastly, the significant association between occurrence of oral lesions with CD4+ counts < 200 cells/mm^3 ^and WHO clinical stage of HIV disease (Table 1) found in this study underscores the prognostic significance of these manifestations in HIV disease.

## Conclusion

In this group of HIV-infected patients, adult patients receiving HAART predominantly combination of stavudine, lamivudine and nevirapine for a period of less than a year, had a significantly lower prevalence of oral lesions, particularly oral candidiasis and oral hairy leukoplakia that was observed. There was also an insignificant increase in prevalence of oral warts and mucosal hyper pigmentation, while the prevalence of other oral lesions was unchanged. However there was no significant change in occurrence of oral lesions in children receiving HAART. More studies preferably longitudinal need to be conducted for longer periods of time in order to get a better picture on the efficacy of HAART in reducing oral lesions in both children and adults in our setting.

## Competing interests

The author(s) declared that they have no competing interests.

## Authors' contributions

All authors were involved in designing the study. OJMH and FM took part in data collection, data handling and preparation of manuscript. MINM, ENMS, MJM, FHM, PEV and AJAM participated in preparation of the manuscript. EK participated in data analysis and preparation of the manuscript. Finally, all authors read and approved the final manuscript.

## Pre-publication history

The pre-publication history for this paper can be accessed here:


